# State of Emergency Medicine in Switzerland: a national profile of emergency departments in 2006

**DOI:** 10.1186/1865-1380-6-23

**Published:** 2013-07-10

**Authors:** Bienvenido Sanchez, Alexandre H Hirzel, Roland Bingisser, Annette Ciurea, Aris Exadaktylos, Beat Lehmann, Hans Matter, Kaspar Meier, Joseph Osterwalder, Robert Sieber, Bertrand Yersin, Carlos A Camargo Jr, Olivier Hugli

**Affiliations:** 1 Emergency Department, Lausanne University Hospital, Lausanne, Switzerland; 2 University of Lausanne, Lausanne, Switzerland; 3 Department of Emergency Medicine, University Hospital, Basel, Switzerland; 4 Emergency Department, Bulach Hospital, Bulach, Switzerland; 5 Emergency Department, University Hospital Inselspital, Bern, Switzerland; 6 Emergency Department, Limmattal Hospital, Schlieren, Switzerland; 7 Anesthesiology Department, Regional Hospital, Ilanz, Switzerland; 8 Emergency Department, Cantonal Hospital, Sankt Gallen, Switzerland; 9 Department of Emergency Medicine, Massachusetts General Hospital, Harvard Medical School, Boston, MA 02114, USA

**Keywords:** Emergency medicine, Emergency medical services, Emergency hospital service, Switzerland

## Abstract

**Background:**

Emergency departments (EDs) are an essential component of any developed health care system. There is, however, no national description of EDs in Switzerland. Our objective was to establish the number and location of EDs, patient visits and flow, medical staff and organization, and capabilities in 2006, as a benchmark before emergency medicine became a subspecialty in Switzerland.

**Methods:**

In 2007, we started to create an inventory of all hospital-based EDs with a preliminary list from the Swiss Society of Emergency and Rescue Medicine that was improved with input from ED physicians nationwide. EDs were eligible if they offered acute care 24 h per day, 7 days per week. Our goal was to have 2006 data from at least 80% of all EDs. The survey was initiated in 2007 and the 80% threshold reached in 2012.

**Results:**

In 2006, Switzerland had a total of 138 hospital-based EDs. The number of ED visits was 1.475 million visits or 20 visits per 100 inhabitants. The median number of visits was 8,806 per year; 25% of EDs admitted 5,000 patients or less, 31% 5,001-10,000 patients, 26% 10,001-20,000 patients, and 17% >20,000 patients per year. Crowding was reported by 84% of EDs with >20,000 visits/year. Residents with limited experience provided care for 77% of visits. Imaging was not immediately available for all patients: standard X-ray within 15 min (70%), non-contrast head CT scan within 15 min (38%), and focused sonography for trauma (70%); 67% of EDs had an intensive care unit within the hospital, and 87% had an operating room always available.

**Conclusions:**

Swiss EDs were significant providers of health care in 2006. Crowding, physicians with limited experience, and the heterogeneity of emergency care capabilities were likely threats to the ubiquitous and consistent delivery of quality emergency care, particularly for time-sensitive conditions. Our survey establishes a benchmark to better understand future improvements in Swiss emergency care.

## Background

**Figure 1:**
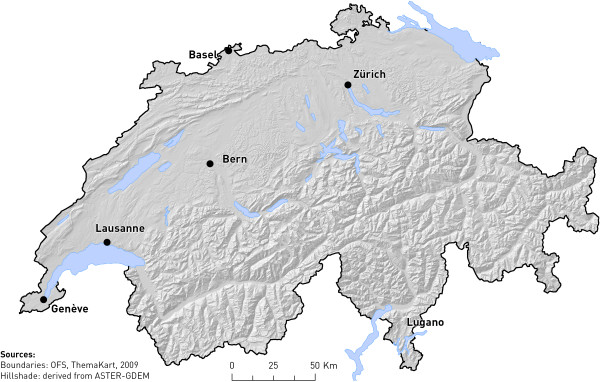
Map of Switzerland.

Switzerland (Figure 1) has joined the growing number of European countries that have either already recognized or are in the process of recognizing emergency medicine as a specialty [1]. Up until now, there has not been a standard for emergency medicine (EM) specialty training programs or a central organization of accreditation that guarantees the quality of EM care and procedures in Switzerland. Moreover, health care politics and the organization of emergency care are governed in a decentralized manner by each of the 26 states, as Switzerland is a federation [2]. Each state delegates emergency care to the emergency departments (ED) of public or private hospitals for which there are currently only non-binding recommendations as to the quality of the organization, training of health care personnel, as well as minimum technical requirements published in 2005 by the Swiss Society of Emergency and Rescue Medicine (SSERM) [3,4]. State of International Emergency Medicine Although emergency medicine is not yet a board-certified specialty in Switzerland [5], it became a subspecialty in 2009 when the Swiss Medical Association required 18 months of additional training to receive a certificate of emergency medicine, above the Swiss Medical Association certificate requirements for general internal medicine, surgery, orthopedic surgery, cardiology, intensive care medicine, or anesthesiology [6]. The training focuses on medical and surgical emergencies of adults only [7]. The first physicians to have undergone this specialized training in emergency medicine were certified in 2011.

Whereas Switzerland, populated with 8 million inhabitants and with four national languages, has a system of public health surpassed by only the USA in per capita costs [8], to this day it does not possess a description of its EDs, an essential component of any health care system. Our objective was to establish the structural characteristics and location, the number of patient visits and flow, and the medical organization and personnel of Swiss EDs in 2006, as a benchmark before emergency medicine became a subspecialty in Switzerland.

## Methods

We defined an ED as any emergency unit linked to a hospital offering acute care 24 h per day, 7 days per week. EDs dedicated solely to ophthalmology or psychiatry were excluded on account of their very specialized structures and technical capabilities. In 2007, we started to create an inventory of all hospital-based EDs with a preliminary list from the SSERM. During 2008 to 2010, while conducting this unprecedented national survey, we improved on this list with input from ED physicians nationwide. For the purpose of our survey, Switzerland was divided into five zones based on linguistics or geography; one or two of the co-authors per zone sent a questionnaire to the head physician of each emergency center, or in the absence of such, to the acting head or to the hospital administration. The contents of the questionnaire were drawn up using structural and organizational recommendations for Swiss hospital EDs as far as the medical and technical aspects were concerned [4]. Other aspects of the questionnaire were drawn from the National ED Inventories (NEDI) project [9,10]. A teaching hospital is defined as a hospital accredited by the Swiss Medical Association for clinical education and training. “Triage to service” refers to the process of patients arriving at the ED being directed to emergency care from non-EM specialists, for example, the medical vs. surgical team. Crowding was defined as more patients than available triage rooms or beds at 6 p.m. on a typical day, while boarding was defined as a wait in the ED >2 h before transfer to the hospital floors by 6 p.m. on a typical day [11]. The list of medical specialties was based on the NEDI list (e.g., general surgeon, anesthesiologist, orthopedic surgeon, obstetrician-gynecologist, cardiologist, pediatrician, psychiatrist, plastic surgeon neurologist, and neurosurgeon), with the addition of two specialties (i.e., internist, and radiologist) of particular importance to EM in Switzerland.

When an ED returned a partially filled questionnaire and we could not elicit another response from the site, either the national report on Swiss hospitals [12], hospital website, or annual reports from individual hospitals were queried for missing data.

For each postal code with a hospital-based ED, we applied one of the 14 Swiss spatial mobility region (SSMR) codes defined in 2000, which were based on the 2000 Swiss census [13]. Briefly, the SSMR codes were developed to measure urbanization and rural character at a regional level, and classifies each of the 106 regions of Switzerland into 14 categories based on complex sets of criteria that include the number of residents, the percentage of commuters, the relative percentage of housing and offices, the proportion of the population engaged in the primary or secondary activity sectors of the economy, as well as the number of overnight tourist stays per resident. The 14 categories are grouped into four levels: (1) metropolitan area, (2) large non-metropolitan urban area with 40,000 to 120,000 residents, (3) small non-metropolitan urban area with <40,000 residents, and (4) rural area [13].

We collected the data describing Swiss EDs and their activities for the period from 1 January 2006 to 31 December 2006. Our goal was to obtain a response rate of 80% of Swiss EDs, a requirement to be part of the NEDI project [10]. Our study was initiated in 2007 and the 80% threshold reached in 2012.

### Statistical analysis

Continuous variables are presented using the average and standard deviation (SD), while data with a non-normal distribution are presented using the median and interquartile range (IQR). Categorical variables are presented as percentages. The results were stratified and analyzed according to the number of visits per year. Inter-group comparisons were done using ANOVA for continuous variables with a normal distribution and the Kruskal-Wallis test for data with a non-normal distribution; categorical data was analyzed with the chi-square test or Fisher’s exact test. Statistical analyses were done using Stata 12.0 (StataCorp, College Station, TX, USA). A two-sided *P* < 0.05 was considered statistically significant.

## Results

**Figure 2:**
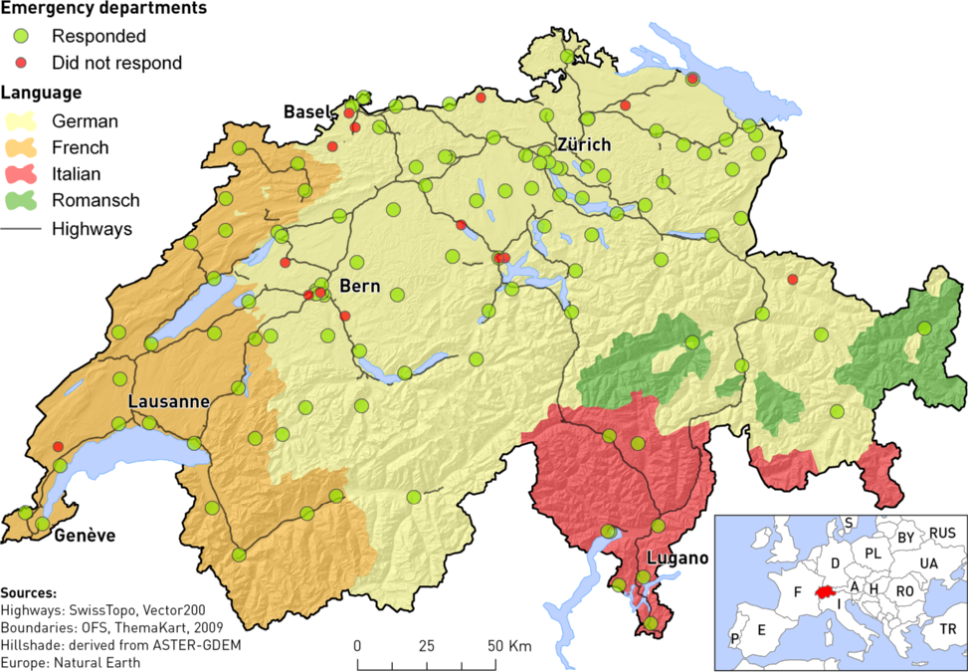
Emergency department locations in Switzerland.

In 2006, Switzerland had a total of 124 hospital-based EDs, or 78 EDs per 10,000 square miles, or 17 EDs per 1,000,000 inhabitants. The total number of EDs that we surveyed was greater than the number of hospitals because some hospitals had separate and autonomous EDs for pediatrics (*n* = 11) or gynecology (*n* = 3) within the same hospital. Therefore, the total ED census was 138; 99 EDs (72%) were located in the German-speaking region of Switzerland, 30 (22%) in the French-speaking region, and 9 (7%) in the Italian-speaking region (Figure 2). Of the 138 EDs, 122 (88%) returned the survey, with 108 (78%) completely filled and 14 (10%) partially filled, encompassing 111 (90%) of the 124 hospitals (Figure 2). By language region, the response rates were 84% German, 97% French, and 100% Italian (*P* = 0.08).

### Number and location of the EDs

**Figure 3:**
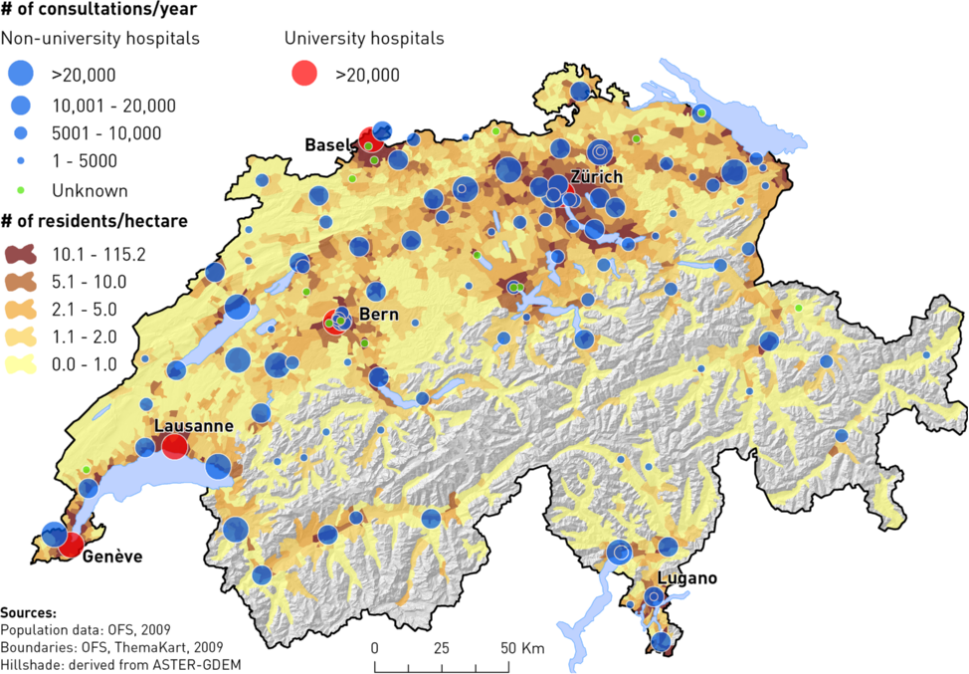
Emergency department visits and population density.

**Figure 4:**
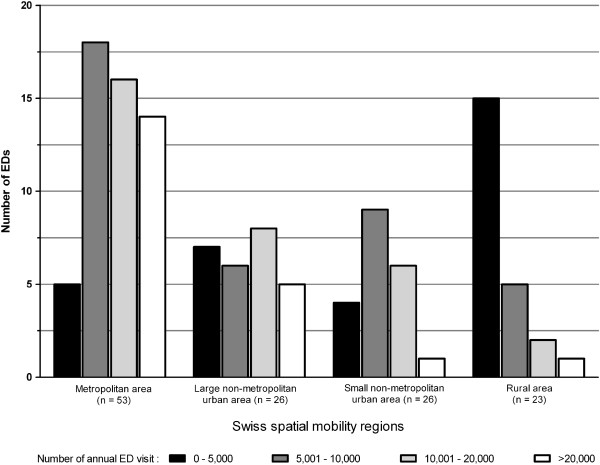
Number of Swiss EDs by urban-rural and annual volumes in 2006.

The vast majority (95%) of EDs were based in teaching hospitals; 9% were in university hospitals, and 87% were at public institutions. The EDs treated both adults and children in 45% of cases, uniquely adults in 46%, uniquely children in 7%, and only gynecology patients in 2%. Most (80%) of all EDs also served as a walk-in clinic*,* providing episodic care to patients without appointment, and for non-urgent conditions. Nearly 2/3 of EDs were located in a metropolitan area or a non-metropolitan city with ≥40,000 residents (Table 1). The EDs were further stratified according to the average number of annual visits: 1 to 5,000 in 31 (25%) of EDs, 5,001 to 10,000 in 38 (31%), 10,001 to 20,000 in 32 (26%), and >20,000 visits in 21 (17%) of EDs (Table 1 and Figure 3). Almost all EDs with >10,000 annual visits were located in an urban setting (Figure 4).

**Table 1:** Number, location of Swiss emergency departments (EDs), and patient flow

**Characteristics**		**Number of visits per year**				
	**All**	**1 – 5,000**	**5,001 - 10000**	**10,001 – 20,000**	**>20,000**	***P value***
Number of EDs, *n* [%]	122	31 [25]	38 [31]	32 [26]	21 [17]	
Urban: – rural status						
Metropolitan area [%]	46	16	47	50	67	
Large non-metropolitan urban area [%]	20	23	16	25	24	*<0.001*
Small non-metropolitan urban area [%]	15	13	24	19	5	
Rural area [%]	19	48	13	6	5	
Total number of visits [thousands]	1,436	89.6	283.4	470.4	631.8	
Median number of visits	8,806	2,595	7,028	15,134	26,317	
[IQR]	[5,196 – 16,180]	[1,742 - 3,909]	[6,086 - 8,607]	[12,063 - 17,000]	[24,621 -32,000]	
Crowding [%]	48	17	32	69	84	*<0.001*
ED capacity estimation :						
Insufficient [%]	41	20	32	62	53	
Adequate [%]	51	64	59	34	47	*0.021*
Underutilized [%]	8	16	9	3	0	
Boarding [%]	48	16	41	66	74	*<0.001*
Hospital capacity estimation:						
Insufficient[%]	29	0	21	48	53	
Adequate [%]	64	92	68	52	42	*<0.001*
Underutilized [%]	7	8	12	0	5	
ICU within hospital	65	23	59	91	95	*<0.001*
ICU capacity estimation:						
Insufficient [%]	31	20	32	26	39	
Adequate [%]	61	60	59	70	50	*0.69*
Underutilized [%]	8	20	9	4	11	

Abbreviations: *IQR: *interquartile range, *ICU: *intensive care unit.

### Patient visits and flow

In 2006, 122 EDs handled 1.475 million visits (Table 1), or 20 visits per 100 inhabitants. The median number of annual visits for each ED was 8,806 (5,196 – 16,180), with a range of 581 to 60,000. Half (50%) of EDs admitted <8,760 patients per year, or less than one patient per hour; of those, 34% were located in a metropolitan area, 20% in a large non-metropolitan urban area, 13% in a small non-metropolitan urban area, and 33% in a rural area (*P* = 0.001).

Overall, 39% of visits were for trauma or surgery, 32% for internal medicine, 15% for pediatrics, and 14% for other medical specialties. Transportation to the ED was estimated to be by ambulance in 15% (IQR 5–25). Crowding was reported at 48% of all EDs, but reached 84% among the larger centers. The ED visit capacity was judged to be insufficient for the demand at 41% of EDs, and once again the insufficiency was more marked at the larger establishments (*P* = 0.02).

After assessment in the ED, 27% (IQR 17–33) of patients were hospitalized in the same hospital, and 2% (IQR 1–5) transferred to a different hospital; 66% of EDs had established protocols to transfer patients to tertiary care hospitals for specialized treatments. Boarding was reported by almost half of all EDs, and by 2/3 of the largest EDs (*P* < 0.001). Two-thirds (65%) of EDs were located in a hospital with an intensive care unit (ICU), but this percentage ranged from 23% in the smallest EDs to 94% in the largest. The capacity for admission to an acute care bed was deemed insufficient by 29% of all EDs and was 53% among the largest EDs; the opposite was true at smaller hospitals, where overcapacity was more prevalent (*P* < 0.001). With regard to capacity in the ICU, this was deemed insufficient in 31% of EDs.

### Medical staff and organization

The majority of EDs (90%) had triage to service. Initial patient evaluation was done by trainees (residents) in 77% of cases and attending physicians in 6%, while the remainder was handled by both (Table 2). These respective percentages varied by the size of the ED (*P* = 0.009). More than half (58%) of all EDs had no requirement for prior post-graduate training for the residents in order to work there; one-third (35%) of EDs required at least 6 months and 7% required at least 24 months. The duration of ED rotation was very short for the most part: ≤3 months for 51% of all EDs with very few exceeding 6 months. The respondents from the smallest EDs favored a rotation >6 months, those from medium-sized EDs favored <3 months, and those from the largest EDs favored a 4 to 6-month rotation (*P* < 0.001). Physicians trained in advanced cardiac life support (ACLS) were present in 89% of EDs, in advanced trauma life support (ATLS) in 82%, and in pediatric life support (PALS) in 56%, but these individuals were not always available within 10 min (Table 2). Only the percentage of PALS-trained physicians was significantly higher in the larger EDs (*P* = 0.01). EDs staffed with physicians providing all three types of advanced life support were also more common in larger EDs (*P* = 0.002).

**Table 2:** Medical staff and organization

**Characteristics**		**Number of visits per year**				
	**All**	**1 – 5,000**	**5,001 – 10,000**	**10,001 – 20,000**	**>20,000**	***P value***
Initial patient care handled by:						
Residents only [%]	77	68	66	97	79	
Attending physicians only [%]	6	16	3	0	5	*0.009*
Both [%]	18	16	31	3	16	
Required post-graduate training for residents before ED rotation:						
None [%]	58	62	70	57	33	
≥ 6 months [%]	35	38	18	39	56	*0.051*
≥ 24 months [%]	7	0	12	4	11	
Duration of ED rotation						
≤ 3 months [%]	51	22	65	69	22	
4–6 months [%]	31	28	18	24	72	*< 0.001*
> 6 months [%]	18	50	18	7	6	
Physician(s) certified in advanced life support:						
ACLS [%]	89	91	85	93	88	*0.83*
Available <10 min [%]	82	87	72	89	78	*0.59*
ATLS [%]	82	76	71	93	89	*0.14*
Available <10 min [%]	71	64	52	89	83	*0.61*
PALS [%]	56	40	52	52	89	*0.011*
Available <10 min [%]	40	29	38	32	80	*0.53*
All 3 certifications [%]	46	23	38	52	82	*0.002*

Abbreviations: *ACLS: *advanced cardiac life support, *ATLS:* advanced trauma life support, *PALS: *pediatric advanced life support.

The daily availability of specialists varied greatly across specialty type, as well as across hospital size (Table 3). Smaller EDs had limited access to specialists in radiology, pediatrics, psychiatry, plastic surgery neurology, and neurosurgery specialists, with very rare access to the latter five (14%, 9%, 8%, 8%, and 12%).

**Table 3:** Daily availability of specialized physicians

**Characteristics**		**Number of visits per year**				
	**All**	**1 – 5,000**	**5,001 – 10,000**	**10,001 – 20,000**	**>20,000**	***P value***
General surgeon [%]	97	95	97	100	95	*0.69*
Anesthesiologist [%]	94	91	97	93	95	*0.80*
Orthopedic surgeon [%]	85	75	87	89	90	*0.55*
Obstetrician-gynecologist [%]	89	83	92	92	89	*0.87*
Cardiologist [%]	58	44	58	54	81	*0.08*
Pediatrician [%]**	56	14	68	53	90	*0.001*
Psychiatrist [%]	45	9	47	54	74	*<0.001*
Plastic surgeon [%]	32	8	33	34	58	*<0.01*
Neurologist [%]	29	8	26	25	68	*<0.001*
Neurosurgeon [%]	23	12	23	18	47	*0.052*
Internist [%]*	97	95	100	92	100	*0.40*
Radiologist [%]	86	65	87	96	89	*0.03*

*Pediatric-only hospitals excluded (*n *= 7).

**Adult-only hospitals excluded (*n *= 45).

### Capabilities

With respect to available technical resources, a resuscitation room was on hand in 77% of EDs, from 56% in small EDs to 95% of the larger centers (*P* = 0.001). The majority of EDs had immediate access to vital laboratory tests, blood gas measurements, and EKGs (Table 4); immediate blood transfusion was available in 53% of EDs and other blood products only in 28%, both of them being similar across ED size.

**Table 4:** Capabilities

**Characteristics**		**Number of visits per year**				
	**All**	**1 – 5,000**	**5,001 – 10,000**	**10,001 – 20,000**	**>20,000**	***P value***
Resuscitation room [%]	77	56	67	93	95	*0.001*
Laboratory tests available:						
Vital laboratory results <30 min [%]	88	92	89	90	79	*0.63*
Blood gases <15 min [%]	98	100	97	100	95	*0.55*
Electrocardiogram <15 min [%]	99	100	97	100	100	*1.0*
Blood products						
Blood transfusion <15 min [%]	53	44	43	66	63	*0.19*
Other blood products <15 min [%]	28	20	29	38	21	*0.49*
Medical imaging:						
X-ray (<15 min), [%]	100 (70)	100 (60)	100 (57)	100 (90)	100 (79)	*-- (0.014)*
CT (non-contrast head CT <15 min), [%]	87 (38)	60(16)	92 (34)	100 (55)	95 (47)	*<0.001 (0.019)*
Ultrasound (<15 min), [%]	97 (56)	88 (72)	100 (51)	100 (59)	100 (42)	*0.016 (0.22)*
FAST immediately available (performed by ED physician), [%]	61 (58)	48 (70)	48 (60)	76 (62)	72 (36)	*0.07 (0.5)*
24/7 availability of:						
Operating room [%]	87	64	91	97	95	*<0.01*
Dialysis [%]	41	8	29	62	74	*<0.001*
Catheterization laboratory [%]	21	8	20	14	48	*0.01*
Stroke unit [%]	19	4	14	21	45	*0.007*

Abbreviations: *FAST *focused assessment with sonography for trauma.

All EDs had access to conventional medical imaging, but access to CT or ultrasound was less common in small EDs (*P* < 0.001 and 0.016, respectively). Only 19% of all had a CT scanner within the ED. Access to a non-contrast head CT or x-ray in <15 min was less common in smaller EDs (*P* = 0.02 and 0.01, respectively). Immediate focused assessment with sonography for trauma (FAST) was available in 61% of EDs and, when available, was performed by ED physicians in 58% of cases. An operating room, dialysis, catheterization laboratory, and a stroke unit available 24/7 were more commonly found in larger EDs (all *P* ≤ 0.01).

## Discussion

Emergency care is uniquely defined by the urgency and location of treatment and transcends the usual boundaries of most organizations and medical specialties [14]. Universal provision of quality emergency care, particularly for time-sensitive conditions (such as fibrinolytic therapy for acute ischemic stroke, revascularization in acute myocardial infarction, or management of severe injury or trauma), is an essential component of any highly developed health care system, such as the Swiss health care system. The delivery of quality emergency care therefore requires physicians with broad expertise and substantial resources. Our national survey, using data from 2006, is the first to offer a global picture of EDs in Switzerland.

We found similarities as well as differences between Swiss EDs and those of other countries. First of all, the 122 Swiss EDs handled approximately 1.475 million visits or 20 visits per 100 inhabitants in 2006, a figure that underestimates the true number since only 88% of EDs responded to our survey. However, based on hospital characteristics associated with the number of ED visits, the 16 non-responding hospitals accounted for approximately 133,000 visits. The total annual number of ED visits was close to 1.5 million, therefore 1.6 times higher than the only other national estimate available (820,000 visits) [15], which was based on a regional sample collected in 2006. Thus, EDs are even more significant contributors to health services in Switzerland than is officially recognized. With respect to the number of inhabitants, the number of ED visits is less than, but close to that of other European countries, such as France and the UK, which each have about 24 visits per 100 inhabitants [16,17]. It is, however, considerably less than the 41 visits per 100 inhabitants in the USA [18], and this despite a higher density of EDs per square mile: 2.9 times higher than in France and 5.6 times higher than in the USA [19]. This discrepancy between ED visits and ED density illustrates that the quote “if you build it they will come” [20] does not always apply, as other factors such a higher physician density mitigate this correlation [21]; the Swiss population has rapid access not just to medical care at an ED within close proximity, but also to a density of office-based physicians that is 1.6 times higher than in the USA [22].

Among Swiss EDs, the activities at the different EDs varied greatly, from underutilized to crowding. Almost 60% of EDs had ≤10,000 annual visits, twice the US proportion [19]. But even more significant is the fact that half of Swiss EDs saw less than one patient per hour. These low-volume EDs typically do not have broad medico-technical resources, specialized staff, or direct access to acute-care specialists. Critical equipment such as a resuscitation or operating room and ICU were available in two-thirds or less of EDs admitting up to 10,000 patients per year. Imaging capabilities such as CT scans were also less common in low-volume EDs, and when present, took longer to obtain. Furthermore, permanent access to radiologists, pediatricians, psychiatrist, neurologists, and neurosurgeons was almost non-existent; the ability of low-volume EDs to manage time-sensitive conditions was therefore limited. This issue is circumvented in part through regionalization [23]. Although each of the independent 26 Swiss cantons has developed its own comprehensive health care system, bi- or multilateral arrangements between neighboring cantons have been implemented to maximize the use of highly specialized care [2], a fact illustrated by existing transfer protocols reported by two-thirds of Swiss EDs, a proportion slightly higher than in the UK [24]. However, transfer of patients to larger facilities may not be an option at any time of the year for EDs located in mountainous areas.

In addition, regionalization often results in centralization, or a one-way transfer from smaller to larger EDs [25], and may exacerbate the crowding of high-volume EDs and their associated hospitals. Crowding threatens the quality and effectiveness of patient care across a variety of medical conditions [26] and is reported by most Swiss EDs. Crowding is often conceptualized as the result of increased demands of ED services (input), suboptimal care processes that prolong the ED stay (throughput), and finally inefficient management or placement of ED patients (output) [27]. Limited data suggest that the number of ED visits has increased significantly over time [28]. Physicians with limited experience in EM who staff Swiss and other European EDs contribute to a slower patient throughput [29]. Finally, an insufficient number of acute inpatient beds, a consequence of the significant reduction of acute care hospital beds since 1990 in Switzerland [22], adversely affects output.

The lack of clinical experience in EM is a concern not just with regard to crowding but also to the quality of care delivered to the most critically ill ED patients. Junior residents may lack the necessary knowledge of EM because of the current deficient undergraduate teaching of acute care medicine in European medical schools [30]. Recent graduates of Swiss medical schools have suboptimal competencies to work up or treat common [31] or critical emergency situations [32]. Their intermittent or short-duration work in the ED during resident training may be too little exposure to EM to solidify their knowledge and skills in this field; this may be further aggravated by the lack of or limited supervision during night and weekend shifts [5]. Recent developments in the status of EM as a subspecialty provide an opportunity for certified Swiss ED physicians in the ED to participate in the undergraduate and postgraduate teaching of EM in Swiss medical schools, both in the lecture hall and at the bedside [33]. We anticipate that the greater involvement of ED physicians in teaching and in the direct care of patients will improve residents’ skills and patient outcomes, an improvement that we hope to demonstrate in future national surveys.

The vast majority of EDs used triage to service, which reflects the absence of EM-trained specialists in this country. This proportion is higher than in other European countries such as Denmark (54%) [34], but closer to others like Slovenia (100%) [35]. A substantial body of literature highlights the benefits of EM-trained physicians for the care of critically ill patients [36,37]. However, a large proportion of Swiss EDs have such a low volume of patients that a dedicated team of ED physicians is not clinically or financially feasible; this problem has also been reported in other countries [38]. Closure of smaller EDs may be an option given the relatively small size of Switzerland, especially for EDs in densely populated areas; however, it may deprive remote areas of their access to emergency care. Previous attempts to close smaller EDs have in fact been met with strong opposition from the voters, and politicians have been forced to withdraw all such proposals in the past [2]. As suggested, one alternative to dedicated ED physicians is to integrate EM core curricula into primary care programs such as internal or family medicine [39]. Another approach is to provide high-quality emergency care through telemedicine, a field not yet developed in Switzerland. Observational studies suggest a beneficial impact of telemedicine for pediatric [40,41], neurosurgery [42], stroke [43], and both major and minor trauma cases [44,45], all of which require medical specialists that are rarely available in smaller Swiss EDs.

Finally, our study highlights the heterogeneity of emergency care capabilities provided by Swiss EDs. Although high-volume EDs provided more comprehensive services in general, there were many exceptions. There is no explicit ED categorization to date, although classification criteria have been published in 2005 [4]. The establishment of a map detailing the available competencies and resources of all Swiss EDs is essential to guide individual patients or Emergency Medical Systems to the appropriate ED; categorization may also provide decision-makers with the information required to plan emergency care at the regional and national level [46,47]. Providing such a list is in fact a requirement for EDs who want to be certified by the SSERM as training programs for EM.

Our study provides the largest, most inclusive perspective on Swiss EDs to date with participation of 88% of all Swiss EDs. Our survey was conducted 6 years ago, a delay explained by the difficulty in creating a complete national list and reaching a response rate of 80%; however, our goal is to provide data that will serve as a benchmark to assess future progress and allow for international comparisons. Another limitation is that data were collected over a 6-year period, and we therefore cannot exclude a recall bias. However, approximately 75% of questionnaires were returned in 2007 and 2008, minimizing the potential magnitude of this issue*.* Our survey did not include any emergency care performance measures, such as those developed in the Physician Quality Reporting Initiative developed by the Centers for Medicare & Medicaid Services (CMS) [48]. Therefore, the quality of care provided in Swiss EDs cannot be inferred from our data, but constitutes a field for future research projects. In one study assessing patients’ experience in a limited number of Swiss EDs, patients reported satisfaction with their care management in the ED [49]. Finally, these data were self-reported, and we could not verify their complete accuracy.

## Conclusion

In 2006, Switzerland had a high density of hospital-based EDs with a significantly larger number of ED visits than previously estimated. Our national survey demonstrates that EDs play a significant role in the delivery of health care for the Swiss population. It also identified crowding and the provision of emergency care by physicians with limited experience in EM as potential threats to the universal delivery of quality emergency care, particularly for time-sensitive conditions. Further studies are needed to assess the quality of care in Swiss EDs. Along those lines, our survey establishes a benchmark at the inception of major changes in Swiss emergency care and will promote a better understanding of the importance and eventual impact of planned improvements in Swiss emergency care.

## Abbreviations

ACLS: Advanced cardiac life support; ATLS: Advanced trauma life support; ED: Emergency department; EM: Emergency medicine; EMNet: Emergency Medicine Network; FAST: Focused assessment with sonography for trauma; IQR: Interquartile range; NEDI: National Emergency Department Inventory; PALS: Pediatric advanced life support; SD: Standard deviation; SSERM: Swiss Society of Emergency and Rescue Medicine.

## Competing interests

The authors declare that they have no competing interests.

## Authors’ contribution

BS, BY, CC, KM, and OH contributed to the creation of the questionnaire; RB, AC, AD, BL, HM, KM, JO, RS, and OH collected the data; AH and OH generated the geographical analyses; OH performed the statistical analyses; all authors critically revised the manuscript. All authors read and approved the final manuscript.
